# Osteomyelitis of the Cervical Spine Presenting as Acute ST-Segment Elevation

**DOI:** 10.7759/cureus.21296

**Published:** 2022-01-16

**Authors:** Charles Meadows, Ameer Quadri, Rodrigo Aguilar, Mehiar Elhamdani

**Affiliations:** 1 Internal Medicine, Marshall University Joan C. Edwards School of Medicine, Huntington, USA; 2 Cardiology, Marshall University Joan C. Edwards School of Medicine, Huntington, USA

**Keywords:** intravenous drug user, st-elevation myocardial infarction (stemi), atypical chest pain, osteomyelitis, myocardial infarction, electrocardiography

## Abstract

Acute ST-segment elevation (STE) on electrocardiogram (EKG) is very frequently associated with myocardial infarction, which requires prompt diagnosis and treatment. However, there are multiple other causes of acute STE, both cardiac and noncardiac. Here we describe a unique case of acute inferior and lateral STE caused by osteomyelitis and abscess of the lower cervical vertebrae.

## Introduction

Acute ST-segment elevation (STE) on electrocardiogram (EKG) is always concerning and may require urgent coronary angiography if STEMI (ST-segment elevation myocardial infarction) is suspected. However, not all patients presenting with STE have acute coronary syndrome (ACS). In addition, some cases of STE have noncardiac etiology. We present a case of a patient with acute inferior and lateral STE caused by osteomyelitis and abscess of the lower cervical vertebrae. While acute STE will always necessitate an early invasive workup in many patients, wider recognition of uncommon causes may help streamline diagnosis in those patients found not to have STEMI.

## Case presentation

**Figure 1:**
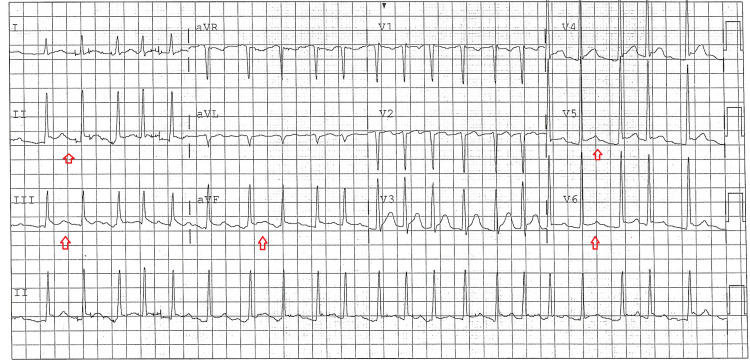
EKG at presentation, showing STE in inferior and lateral leads (red arrows). EKG: electrocardiogram; STE: ST elevation.

**Figure 2:**
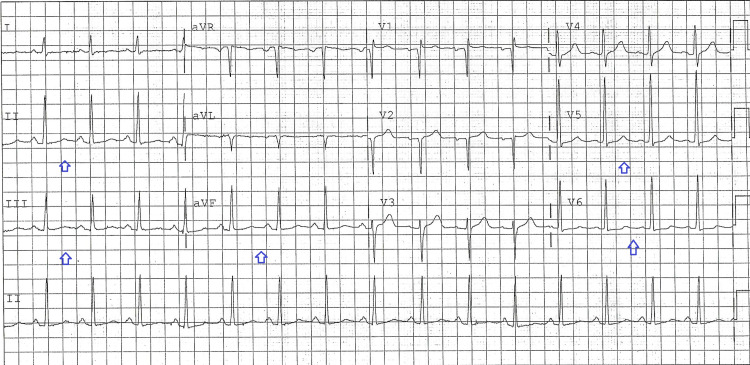
EKG at follow-up, showing resolution of inferolateral STE (blue arrows). EKG: electrocardiogram; STE: ST elevation

A 47-year-old male presented to the emergency room with sharp chest pain of four days duration, associated with right arm numbness. Past medical history was positive for hypertension, injection drug use (last use three days prior), and cigarette smoking. In the ED, vitals were remarkable only for mild tachycardia, with blood pressure 132/87, heart rate 106, respiratory rate 18, oxygen saturation 98%. The patient was afebrile at 98.5. On examination, lungs were clear and the heart was irregular with no extra sounds. Some skin changes consistent with superficial phlebitis were present in the right forearm. Chest X-ray showed no cardiopulmonary disease. EKG showed atrial fibrillation with rate 136 and 2mm ST elevation in leads II, III, aVF as well as in the low lateral leads (Figure 1). On the basis of his chest pain and EKG findings, the patient was taken for emergent coronary angiography, which showed no angiographic evidence of underlying coronary artery disease or acute coronary thrombosis. Ejection fraction was 55% with no wall motion abnormalities. Initial chemistry (not available at the time of coronary angiography) was remarkable for sodium 128 mmol/L, serum creatinine 1.5 mg/dL, and lactic acid 3.0 mmol/L. White blood count was 23,000/microliter with hemoglobin 12.0 mg/dL and platelets 176,000/microliter. Initial high sensitivity troponin I was 19.8 pg/ml (normal 3-58) and NT Pro-BNP was 1032 pg/ml (normal 5-125). Labs are presented below in Table 1. Computed tomography (CT) chest was unremarkable. Cardiology recommended workup for nonischemic causes of chest pain, including possible pericarditis. Given the lab results concerning for acute infection, the patient was started on broad-spectrum antimicrobials and admitted to the medical service. CT neck was ordered (based on the patient's report of arm tingling) which showed phlegmonous changes of the cervical spine with anterior cervical and paraspinal abscesses. These findings were confirmed on magnetic resonance imaging (MRI). The patient underwent surgical debridement and was admitted to the intensive care unit. An echocardiogram was performed the following day which was unremarkable. The patient was eventually discharged home on appropriate antibiotics but returned for evaluation five weeks later after a fall. At that time all EKG changes, including the atrial fibrillation, had resolved (Figure 2).

**Table 1:** Laboratory results NT-proBNP: N-terminal pro-brain natriuretic peptide

Labs	Result	Normal range
Sodium	128 mmol/L	135-145 mmol/L
Creatinine	1.5 mg/dL	0.6-1.3 mg/dL
Lactic acid	3.0 mmol/L	0.4-2.0 mmol/L
White blood cell count	23.0 thousand/microliter	4.5-10 thousand/microliter
Hemoglobin	12.0 mg/dL	14.0-16.0 mg/dL
Platelets	176 thousand/microliter	150-440 thousand/microliter
Troponin I (high sensitivity)	19.8 pg/mL	3-58 pg/mL
NT pro BNP	1032 pg/mL	5-125 pg/mL

## Discussion

Given that myocardial infarction is the most common cause of acute STE in patients with acute chest pain, emergent coronary angiography is often performed. Still, other important causes (cardiac and noncardiac) remain. One study found that 2.3% of such patients had an etiology for STE unrelated to cardiac ischemia [1]. While cardiac causes such as pericarditis, myocarditis, and coronary vasospasm are well documented and their mechanisms fairly well-understood, less is known about noncardiac causes of STE. Severe intraabdominal pathology has been associated with STE, with acute pancreatitis being the best described [2]. Acute gastritis [3] and small bowel obstruction [4] have also been described in case reports. In none of these cases however has a clear demonstration of the direct cause of STE been elucidated. Electrolyte disturbances have been suggested as a potential mechanism [2], as has compression of the pericardial tissue [4]. Most STE from intra-abdominal pathology occurs in the inferior leads, leading to speculation that changes in vagal tone may be involved [5]. In addition, Takotsubo cardiomyopathy, a condition of apical ballooning secondary to stress-induced sympathetic overstimulation, often associated with STE, has been reported in the setting of acute pancreatitis [6]. To our knowledge, this is the first report of acute STE associated with anterior cervical abscess. Local compression (with possible irritation) of the pericardium has been postulated as a cause of STE in cases of hiatal hernia [7] and esophageal balloon tamponade [8]. In our patient, neither echocardiogram nor MRI neck showed any clear pericardial fluid or inflammation, but local irritation nevertheless seems a possible cause. Pericarditis must surely still be considered in this patient, but the lack of findings on physical exam and echocardiography mitigate against this, as does the lack of global STE on EKG. In addition, acute STE (including STEMI) has been described in sepsis, often associated with elevated troponin levels [9]. We think this is less likely in our patient given the lack of reciprocal changes on EKG as well as his normal serum troponin and coronary angiography. Several recent papers in the electrophysiology literature have examined the molecular basis for STE [10,11]. Multiple simultaneous processes appear to be involved. These include diastolic and systolic currents of injury as well as intramural and transmural conduction delays. In addition, there appears to be a more pronounced effect on the epicardial action potential dome and amplitude [11]. With spinal infections increasing in frequency with more people injecting drugs, more such cases may occur [12].

## Conclusions

Our patient had resolution of his STE with the treatment of osteomyelitis, suggesting inflammation as a primary cause. Given the complexity of the processes involved it may, at this time, be imprudent to proffer anything more specific than an observation that any local inflammatory or compressive processes seem capable of causing nonischemic STE. To our knowledge, this is the first reported case of cervical osteomyelitis presenting as acute STE. Acute STE will more often than not require invasive workup. Still, we hope this study will contribute to the understanding of noncardiac pathology presenting as acute STE. Finally, we hope that this study will highlight the increasing frequency and scope of severe disease associated with injection drug use, and underlying opioid addiction.
